# Multi-choice stochastic transportation problem involving general form of distributions

**DOI:** 10.1186/2193-1801-3-565

**Published:** 2014-09-27

**Authors:** Abdul Quddoos, Md Gulzar ull Hasan, Mohammad Masood Khalid

**Affiliations:** Department of Statistics and Operations Research AMU, Aligarh, India

**Keywords:** General form of distributions, Multi-choice programming, Stochastic transportation problem, Transformation technique

## Abstract

Many authors have presented studies of multi-choice stochastic transportation problem (MCSTP) where availability and demand parameters follow a particular probability distribution (such as exponential, weibull, cauchy or extreme value). In this paper an MCSTP is considered where availability and demand parameters follow general form of distribution and a generalized equivalent deterministic model (GMCSTP) of MCSTP is obtained. It is also shown that all previous models obtained by different authors can be deduced with the help of GMCSTP. MCSTP with pareto, power function or burr-XII distributions are also considered and equivalent deterministic models are obtained. To illustrate the proposed model two numerical examples are presented and solved using LINGO 13.0 software package.

## 1 Introduction

The transportation problem is one of the oldest applications of Linear Programming Problem (LPP). The standard form of the transportation problem was first formulated along with the constructive method of solution by Hitchcock (1941). In a classical transportation problem, a product is to be transported from *m* sources to *n* destinations. The availability of the product at *i*^*th*^ source is denoted by *a*_*i*_, where *i*=1,2,…,*m* and the demand required at *j*^*th*^ destination is *b*_*j*_ where *j*=1,2,…,*n*. The penalty *c*_*ij*_ is the cost coefficient of the objective function which can represent transportation cost, delivery time etc. In many real world situations the availability *a*_*i*_ and demand *b*_*j*_ are not certainly known to Decision Maker (DM). One way to deal such uncertainty is to describe the availability *a*_*i*_ and demand *b*_*j*_ parameters as random variables rather than the deterministic one. These random variables *a*_*i*_ and *b*_*j*_ are assumed to follow a given probability distribution or its probability distribution may be estimated. This type of transportation problem is known as “Stochastic Transportation Problem” (STP). Furthermore, suppose that there exist *k* routes for transporting the product from *i*^*th*^ source to *j*^*th*^ destination and the cost of transporting a unit of product via *k*^*th*^ route is denoted by . Thus DM have multiple (*i.e.* ‘*k*’) route choices for shipping the product from *i*^*th*^ source to *j*^*th*^ destination and he has to identify exactly one among *k* routes in such a manner that the combination of choices should minimize the overall transportation cost. With the above discussed objective the STP becomes ‘Multi Choice Stochastic Transportation Problem’ (MCSTP) in which the cost coefficient *C*_*ij*_ are multi-choice and availability *a*_*i*_ and demand *b*_*j*_ are random variables.

MCSTP has been extensively studied by many researchers. Roy et al. (2012) presented an equivalent deterministic model of MCSTP by assuming that both availability *a*_*i*_ and demand *b*_*j*_ as random variables following exponential distribution. Biswal and Samal (2013) obtained an equivalent deterministic model of MCSTP in which they considered that both *a*_*i*_ and *b*_*j*_ follow Cauchy distribution. Mahapatra (2014) also given equivalent deterministic model of MCSTP involving Weibull distribution. Mahapatra et al. (2013) considered the MCSTP involving Extreme value distribution. Barik et al. (2011) presented a stochastic transportation model involving Pareto distribution.

These random variables *a*_*i*_ and *b*_*j*_ may also be considered to follow Burr-XII or Power Function distributions. Burr-XII may be used in place of normal distribution when data shows some positive skewness. Since *a*_*i*_ and *b*_*j*_ are the physical quantities so it is advisable to use Burr-XII instead of Normal distribution. When upper bound of availability and demand is known, Power Function distribution would be most suitable distribution to fit. In this paper we considered general form of MCSTP, where *a*_*i*_ and *b*_*j*_ are assumed to follow ‘General classes of distribution’ and obtained a generalised equivalent deterministic model (GMCSTP). All the models discussed above by many authors have been deduced by using the proposed GMCSTP. Three new equivalent deterministic models of MCSTP have also been obtained by considering that both *a*_*i*_ and *b*_*j*_ follow Pareto, Power Function and Burr-XII distribution (only one at a time). An equivalent deterministic GMCSTP has also been obtained by considering that *a*_*i*_ follows any one distribution among Exponential, Weibull, Cauchy, Exterme Value, Pareto, Power Function or Burr-XII and *b*_*j*_ follows any other distribution except that of distribution of *a*_*i*_. To illustrate the proposed models two numerical examples are taken and solved by using transformation technique given by Biswal and Acharya (2009). Lingo 13.0 software has been used for obtaining the optimal solution.

## 2 General classes of distributions

Let us consider a random variable *y* following any of the two general classes of distributions with distribution function (*df*)*F*(*y*) as follows:
2.1

and
2.2

where *h*(*y*) is a monotonic and differentiable function of *y* and *p*,*q*,*r* and *h*(*y*) are chosen such that *F*(*y*) in (2.1) and (2.2) are *df* over (*ξ*,*ϕ*).

Differentiating (2.1) and (2.2) with respect to *y* the probability density function (*pdf*), *f*(*y*) may be obtained respectively as,
2.32.4

where *F*(*ξ*)=0 and *F*(*ϕ*)=1.

## 3 Mathematical model of multi-choice stochastic transportation problem (MCSTP)

In this section a mathematical model of multi-choice transportation problem involving general form of distributions (2.1 or 2.2) is considered. The general form of MCSTP is:

**MCSTP1:**3.1

subject to,
3.23.33.4

where 0<*α*_*i*_<1, ∀*i* and 0<*β*_*j*_<1, ∀*j*, are the aspiration levels.

It is assumed that *a*_*i*_, *i*=1,2,…,*m*, *b*_*j*_, *j*=1,2,…,*n* are random variables following general form of distribution,  are multi-choice parameters and *x*_*ij*_ are deterministic decision variables.

The following cases are to be considered:

(i) Only *a*_*i*_, *i*=1,2,…,*m* follows general form of distribution.
(ii) Only *b*_*j*_, *j*=1,2,…,*n* follows general form of distribution.
(iii) Both *a*_*i*_, *i*=1,2,…,*m* and *b*_*j*_, *j*=1,2,…,*n* follow general form of distribution.

### 3.1 Only ***a***_***i***_***i=1,2,…,m***follows (2.1) or (2.2)

It is considered that *a*_*i*_, *i*=1,2,…,*m* are independent random variable which follows any of two general form of distributions as defined in (2.1) and (2.2) consider the probabilistic constraint (3.2),


*or*3.5

the above inequality (3.5) can be represented as
3.6

Thus, we obtained a multi-choice deterministic model **MCSTP 2** as follows:

**MCSTP 2:**3.7

subject to,
3.83.93.10

where  (feasibility condition).

### 3.2 Only ***b***_***j***_***, j=1,2,…,n***follows (2.1) or (2.2)

It is considered that *b*_*j*_, *i*=1,2,…,*n* are independent random variable which follows any of two general form of distributions as defined in (2.1) and (2.2) consider the probabilistic constraint (3.3),
3.11

the above inequality (3.11) can be represented as
3.12

Thus, we obtained a multi-choice deterministic model **MCSTP 3** as follows:

**MCSTP 3:**3.13

subject to,
3.143.153.16

where  (feasibility condition).

### 3.3 Both ***a***_***i***_***(i=1,2,…,m)***and ***b***_***j***_***(j=1,2,…,n)***follow (2.1) or (2.2)

It is considered that *a*_*i*_ (*i*=1,2,…,*m*) and *b*_*j*_, *j*=1,2,…,*n* are independent random variable which follows any of two general form of distributions as defined in (2.1) and (2.2).

In view of (3.6) and (3.12) we may obtain a multi-choice deterministic model **GMCSTP** as follows:

**GMCSTP:**3.17

subject to,
3.183.193.20

where  (feasibility condition).

## 4 Different cases of GMCSTP

Consider the following three cases of **GMCSTP**

- when *a*_*i*_ and *b*_*j*_ both follow general form of distribution defined in (2.1).
- when *a*_*i*_ and *b*_*j*_ both follow general form of distribution defined in (2.2).
- when *a*_*i*_ and *b*_*j*_ follow general form of distribution defined in (2.1) and (2.2) respectively or vice-versa.

### 4.1 When ***a***_***i***_and ***b***_***j***_both follow general form of distribution defined in (2.1)

Let us consider that *a*_*i*_ and *b*_*j*_ follows general form of distribution of the form defined in (2.1) .

Putting  in (3.18) of **GMCSTP** and  in (3.19) of **GMCSTP**, we get,

**GMCSTP 1:**4.1

subject to,
4.24.34.4

### 4.2 When ***a***_***i***_and ***b***_***j***_both follow general form of distribution defined in (2.2)

Let us consider that *a*_*i*_ and *b*_*j*_ in (2.2) .

Putting  in (3.18) of **GMCSTP** and  in (3.19) of **GMCSTP**, we get,

**GMCSTP 2:**4.5

subject to,
4.64.74.8

### 4.3 When ***a***_***i***_and ***b***_***j***_follow general form of distribution defined in (2.1) and (2.2) respectively or vice-versa

Consider a case when *a*_*i*_ follows any one of general form of distributions defined in (2.1) and (2.2) and *b*_*j*_ follows any one of general form of distribution defined in (2.2) and (2.1) respectively, then in view of **GMCSTP 1** and **GMCSTP 2** we have,

**GMCSTP 3:**4.9

subject to,
4.104.114.12

## 5 Deduction of some previous results along with some new results

In this section we deduce some previous results with the help of GMCSTP 1 and GMCSTP 2. Since GMCSTP 1 and 2 has been modelled with the assumption that both *a*_*i*_ and *b*_*j*_ are random variable. So we are considering only GMCSTP 1 and 2 throughout the paper. One can also consider MCSTP 2 or/and MCSTP 3 according to requirement. Many previous models proposed by Roy et al. (2012), Mahapatra (2014), Biswal and Samal (2013) and Mahapatra et al. (2013) can be deduced from **GMCSTP 1** and **GMCSTP 2** by setting different values of .

### 5.1 Deductions using GMCSTP 1 and GMCSTP 2

#### 5.1.1 When ***a***_***i***_and ***b***_***j***_follows exponential distribution

Let us consider that both *a*_*i*_ and *b*_*j*_ follow exponential distribution. In order to deduce the model obtained by S.K. Roy *et al*, we set  and  in **GMCSTP 1** and get,

**MCSTP 4:**5.1

subject to,
5.25.35.4

where  (feasibility condition) and *a*_*i*_≥0,*b*_*j*_≥0 and {*θ*_*i*_,*θj*′}>0 are the parameters of exponential distribution. The above **MCSTP 4** is same as obtained by Roy et al. (2012).

#### 5.1.2 When ***a***_***i***_and ***b***_***j***_follows Weibull distribution

Mahapatra (2014) presented a model by considering both *a*_*i*_ and *b*_*j*_ follow weibull distribution which can be obtained by setting  and  in **GMCSTP 1**, as follows:

**MCSTP 5:**5.5

subject to:
5.65.75.8

where  (feasibility condition) and *a*_*i*_≥0,*b*_*j*_≥0 and {*γ*_*i*_,*γj*′}>0 and {*δ*_*i*_,*δj*′}>0 are shape and scale parameters.

The above **MCSTP 5** is same as obtained by Mahapatra (2014).

#### 5.1.3 When ***a***_***i***_and ***b***_***j***_follows Cauchy distribution

Biswal and Samal (2013) proposed MCSTP model by considering that *a*_*i*_ and *b*_*j*_ follow Cauchy distribution. On Setting  and  in **GMCSTP 1**, we get,

**MCSTP 6:**5.9

subject to:
5.105.115.12

where  (feasibility condition) and −*∞*<*a*_*i*_<+*∞*, −*∞*<*b*_*j*_<+*∞* and  and  are the location and scale parameter of *a*_*i*_ and *b*_*j*_, respectively, Which is a multi-choice approach of the model proposed by Biswal and Samal (2013).

#### 5.1.4 When ***a***_***i***_and ***b***_***j***_follows extreme value distribution

Setting  and  in **GMCSTP 2** it deduces to,

**MCSTP 7:**5.13

subject to:
5.145.155.16

where  (feasibility condition) and −*∞*<*a*_*i*_<+*∞*, −*∞* < *b*_*j*_<+*∞* and {*γ*_*i*_,*γj*′}>0 and {*δ*_*i*_,*δj*′}>0 are location and scale parameters of extreme value distribution, which is same as obtained by Mahapatra et al. (2013).

### 5.2 Some new results using GMCSTP 1, GMCSTP 2 and GMCSTP 3

#### 5.2.1 When ***a***_***i***_and ***b***_***j***_follow Pareto distribution

Let us consider the MCSTP in which *a*_*i*_ and *b*_*j*_ follow Pareto distribution. By setting  and  in **GMCSTP 1**, we get,

**MCSTP 8:**5.17

subject to:
5.185.195.20

where  (feasibility condition) and {*d*_*i*_,*dj*′}>0 and {*θ*_*i*_,*θj*′}>0 are scale and shape parameters respectively and *a*_*i*_≥*d*_*i*_ and *b*_*j*_≥*dj*′.

#### 5.2.2 When ***a***_***i***_and ***b***_***j***_follow Burr-XII distribution

Setting  and  in **GMCSTP 1** we get,

**MCSTP 9:**5.21

subject to:
5.225.235.24

where  (feasibility condition) and *a*_*i*_≥0,*b*_*j*_≥0 and {*θ*_*i*_,*θj*′}>0 and {*k*_*i*_,*kj*′}>0 are shape parameters of Burr-XII distribution.

#### 5.2.3 When ***a***_***i***_and ***b***_***j***_follow power function distribution

Setting  and  in **GMCSTP 2**, we get,

**MCSTP 10:**5.25

subject to:
5.265.275.28

where  (feasibility condition) and {*d*_*i*_,*dj*′}>0 and {*θ*_*i*_,*θj*′}>0 are the scale and shape parameters of *a*_*i*_≥0 and *b*_*j*_≥0 respectively.

#### 5.2.4 When ***a***_***i***_follows Burr XII distribution and ***b***_***j***_follows Extreme value distribution

Setting  and  in **GMCSTP 3** we get,

**MCSTP 11:**5.29

Subject to:
5.305.315.32

where .

#### 5.2.5 When ***a***_***i***_follows power function distribution and ***b***_***j***_follows Pareto distribution

Setting  and  in **GMCSTP 3**.

**MCSTP 12:**5.33

subject to:
5.345.355.36

where  (feasibility condition).

## 6 Numerical illustrations

We consider the numerical example taken by (Mahapatra et al. 2013). Data for multi-choice cost  are appended below in Table 1.

**Table 1:** **Multi-choice transportation cost for route** ***x***_***ij***_

SI. no.	Route: ***x***_***ij***_	Transportation cost(in Rupees)
		per unit (1 unit = 10 kg)
1	(1, 1): *x*_11_	10 or 11 or 12
2	(1, 2): *x*_12_	15 or 16
3	(1, 3): *x*_13_	21 or 22 or 23 or 24
4	(1, 4): *x*_14_	21 or 23 or 25
5	(2, 1): *x*_21_	15 or 17 or 19 or 21 or 23 or 25
6	(2, 2): *x*_22_	10 or 12 or 14 or 16 or 18 or 20
7	(2, 3): *x*_23_	9 or 10 or 11
8	(2, 4): *x*_24_	18 or 19
9	(3, 1): *x*_31_	20 or 21 or 22 or 23 or 24 or 25 or 26
10	(3, 2): *x*_32_	10 or 11 or 12 or 13 or 14 or 15 or 16 or 17
11	(3, 3): *x*_33_	20 or 22 or 25
12	(3, 4): *x*_34_	15 or 20

### 6.1 Illustration 1

Let us consider that we have three known parameters of availability *a*_1_,*a*_2_,*a*_3_ follow Burr-XII distribution. The specified probability levels and shape parameters of *a*_1_,*a*_2_,*a*_3_ are given in Table 2.

**Table 2:** **Specified probability levels and shape parameters of** ***a***_***i***_

Random	Specified	Shape	Shape
parameters***a***_***i***_	probability levels	parameters 1	parameters 2
*a*_1_	0.01	0.002	0.73
*a*_2_	0.02	0.004	0.76
*a*_3_	0.03	0.006	0.79

Further, consider that we have four known parameters of demand *b*_1_,*b*_2_,*b*_3_,*b*_4_ follow extreme value distribution. The specified probability levels and location and scale parameters of *b*_1_,*b*_2_,*b*_3_,*b*_4_ are given in Table 3.

**Table 3:** **Specified probability levels,location and scale parameters of** ***b***_***j***_

Random	Specified	Location	Scale
parameters***b***_***j***_	probability levels	parameters	parameters
*b*_1_	0.04	600	5
*b*_2_	0.05	500	4
*b*_3_	0.06	400	3
*b*_4_	0.07	300	2

Using the data provided in Tables 1, 2 and 3 the following equivalent multi-choice deterministic transportation problem is formulated with the help of GMCSTP 3 as:


subject to,
6.16.26.36.46.56.66.7

Now using the transformation technique proposed by Biswal and Acharya (2009), we obtain the following multi-choice deterministic transportation problem:


subject to, (6.1)-(6.7) where,


The above non-linear mixed integer programming problem is solved by using LINGO 13.0 software package and the optimal solution is obtained as: *x*_11_=615.9927,*x*_22_=382.1037,*x*_23_=408.3469,*x*_32_=129.7771,*x*_34_=305.2464 and rest of the *x*_*ij*_ are zero. The minimum transportation cost is 19532.56 obtained by choosing multi-choice cost as follows:


### 6.2 Illustration 2

Again consider, in the above **illustration 1** the availability *a*_1_,*a*_2_,*a*_3_ are supposed to follow Power Function distribution and the demand *b*_1_,*b*_2_,*b*_3_,*b*_4_ are assumed to follow Pareto distribution. The specified probability levels, scale and shape parameters of *a*_1_,*a*_2_,*a*_3_ are given in Table 4 and of *b*_1_,*b*_2_,*b*_3_,*b*_4_ are given in Table 5 respectively.

**Table 4:** **Specified probability levels, scale and shape parameters of** ***a***_***i***_

Random	Specified	Scale	Shape
parameters***a***_***i***_	probability levels	parameters	parameters
*a*_1_	0.01	1000	100
*a*_2_	0.02	800	70
*a*_3_	0.03	700	60

**Table 5:** **Specified probability levels, scale and shape parameters of** ***b***_***j***_

Random	Specified	Scale	Shape
parameters***b***_***j***_	probability levels	parameters	parameters
*b*_1_	0.04	350	5
*b*_2_	0.05	300	6
*b*_3_	0.06	270	7
*b*_4_	0.07	230	8

Solving this in the similar manner optimal solutions are obtained as *x*_11_=666.2789, *x*_22_=352.9524, *x*_23_=403.5651, *x*_32_=141.3123, *x*_34_=320.6938 and rest of the *x*_*ij*_ are zero. The minimum transportation cost is 20047.93 obtained by choosing multi-choice cost as follows:


## 7 Conclusion

In this paper we have considered a MCSTP where cost coefficient of objective function are assumed to be of multi-choice type and random availability and demand of product are assumed to follow general form of distributions. With this generalized formulation of MCSTP the DM becomes capable to fit any distribution among exponential, weibull, cauchy, extreme value, power function, burr-XII and pareto according to the nature of data. Thus the present model can be applied in several situations of transportation problems when demand and availability are restricted to follow a particular probability distribution. Here, only upto eight choices of multi-choice cost parameters are considered because we were much concerned about random parameters. In further studies extended multi-choice parameters may also be taken into account.
